# Postnatal instead of normally-timed cervical screening (PINCS-1): a protocol for a feasibility study of paired-sample cervical screening and urine self-sampling at 6 weeks and 12 weeks postnatal in the UK

**DOI:** 10.1136/bmjopen-2024-092701

**Published:** 2025-05-30

**Authors:** Victoria Cullimore, Rebecca Newhouse, Holly Baker-Rand, Adam R Brentnall, Kim Chu, Karin Denton, Lorna McWilliams, Alex Sargent, Sudha Sundar, Emma J Crosbie, Jo Morrison

**Affiliations:** 1 Department of Gynaecological Oncology, Somerset NHS Foundation Trust, Taunton, UK; 2 Faculty of Health and Life Sciences, University of Exeter, Exeter, UK; 3 Department of Gynaecological Oncology, University Hospital Southampton NHS Foundation Trust, Southampton, UK; 4 Cancer Prevention & Early Detection Research, NIHR Manchester Biomedical Research Centre, The University of Manchester Faculty of Biology Medicine and Health, Manchester, UK; 5 Wolfson Institute of Population Health, Queen Mary University of London, London, UK; 6 Cellular Pathology, North Bristol NHS Trust, Southmead Hospital, Bristol, UK; 7 Manchester Centre for Health Psychology, Division of Psychology & Mental Health, School of Health Sciences, The University of Manchester, Manchester, UK; 8 Cytology Department, Clinical Sciences Centre, Manchester University NHS Foundation Trust, Manchester, UK; 9 Cancer Sciences, University of Birmingham, Birmingham, UK

**Keywords:** Postpartum Women, CYTOPATHOLOGY, HPV Infection, Colposcopy, Gynaecological oncology

## Abstract

**Introduction:**

Cervical screening rates in the UK are falling, limiting our ability to prevent cervical cancer. Peak incidence of cervical cancer coincides with average age of childbirth, and women with young children are less likely to be screened. Current UK guidelines advise waiting 12 weeks after delivery to perform cervical screening, but this recommendation is not based on evidence from the era of liquid-based cytology or high-risk human papillomavirus (HPV) testing. New mums suggested offering cervical screening at 6 weeks postdelivery, in conjunction with the postnatal check-up with the general practice team in primary care. This study aims to assess the feasibility and acceptability of a paired-sample study design for cervical screening at 6 weeks and 12 weeks postnatal.

**Methods and analysis:**

A study of 100 participants will be performed to assess feasibility and acceptability of cervical screening at both 6 weeks and 12 weeks postnatal, with urine self-sampling using a Colli-pee collection device at each time point. This will inform whether women are prepared to undergo cervical screening at 6 weeks postnatal and the feasibility of a future pair-wise diagnostic test accuracy (of HPV and abnormal cervical cytology) study or whether alternative study designs are needed. Participants must be aged 24.5–64 years old and eligible for the National Health Service Cervical Screening Programme (NHS CSP). At each appointment, participants will complete a questionnaire about their experience and thoughts regarding screening. Substudies ask participants who withdraw or decline to participate their reasons, to identify barriers. The study will be closed for recruitment once 100 participants have completed the 6-week screen in Postnatal Instead of Normally-Timed Cervical Screening (PINCS-1) or if recruitment is poor and 50% not recruited by 6 months, indicating that a paired-sample design is not feasible.

**Ethics and dissemination:**

Ethical approval for PINCS-1 was given by the Stanmore Research Ethics Committee. The results, including participant feedback at each stage, built into the trial design, will inform the design of large studies to determine accuracy and clinical impact of cervical screening at 6 weeks postnatal, identifying whether giving choice (eg, from timing of appointments and/or offering self-sampling) will improve screening uptake. Data will inform the sample size needed for future studies to have adequate power. Results will also inform future NHS CSP management. Results will be shared via scientific publication and via conventional and social media channels accessed by young women.

**Trial registration number:**

ISRCTN10071810.

**STRENGTHS AND LIMITATIONS OF THIS STUDY**

- To our knowledge, this is the first study to focus on acceptability and reliability of cervical screening, including urine self-sampling, in postnatal women, to test hypothesis and generate data to inform further study design, following recommendations by Elridge *et al*.
- There are multiple points at which acceptability will be assessed by collecting participants’ views and participant-reported outcomes.
- Data collection tools have been developed using participant responses in the Attitudes to Postnatal Instead of Normally-Timed Cervical Screening (pre-PINCS) study, to ensure applicability to the postnatal population.
- Pilot diagnostic test accuracy data will inform the sample size calculation for future studies.
- Screening will be performed in secondary care throughout this study, as this study is designed to test the feasibility of a future paired sample diagnostic test accuracy study, not the effect on uptake in a primary care setting; this is a separate question, requiring a different study design.

## Introduction

 Cervical cancer is one of the most preventable malignancies encountered worldwide, due to a combination of primary prevention (human pHuman Papillomavirus (HPV) vaccination) and secondary prevention (cervical screening) strategies. Global elimination of cervical cancer is a key WHO strategy.^1 2^ By 2022, cervical screening coverage rates in England had fallen to 66% in women/people with a cervix aged 25–49 years, and to less than 50% in some areas. This is markedly below the National Health Service Cervical Screening Programme (NHS CSP) standard of 80%. The majority of cervical cancers now occur in underscreened women.^3^,^5^ Women with young children under 5 years of age are less likely to participate in cervical screening, as are individuals from ethnic minority backgrounds and lower socioeconomic groups, and these groups are also more likely to have had more children and at a younger age.^6^

Peak incidence of cervical cancer in the UK between 2016 and 2019 was in the 30–34 year-old cohort, followed by cases in women aged 25–29 years.^7^ This coincides with the average age of mothers giving birth in England and Wales of 30.9 years.^8^ Our local cervical cancer audit between 2016 and 2017 identified that 15% of women diagnosed with cervical cancer were currently or had recently been pregnant and had been eligible for cervical screening in pregnancy or postnatally, but none had attended. We found that 50% of women were overdue for cervical screening by the end of their pregnancy, and by 6 months postnatal, more than half had still not attended screening.^9^ This quality improvement (QI) project included canvassed views of new mothers/parents and primary care providers, through focus groups, which identified causes of poor uptake and generated ideas for change.^9^ One idea, from both new mothers and primary care practice staff, was to offer postnatal cervical screening at the 6-week postnatal check-up, facilitating easier attendance for women by reducing barriers.^10^ Self-sampling for high-risk HPV (hrHPV) was also suggested to improve screening uptake. Interestingly, offering opportunistic vaginal self-sampling at a General Practitioner (GP)/primary care appointment was demonstrated to be an effective strategy in the recent YouScreen study, potentially leading to a 7.6% improvement in overall screening rates.^11 12^

There are numerous barriers to screening in young women, including a perception that this age group is not at risk, inadequate knowledge, and fear of pain, discomfort and embarrassment. However, being busy and not getting around to having a test were independent factors, regardless of screening status.^13 14^ Our work showed that we could improve uptake by 8% in the postnatal cohort, largely by improving education of midwives and women in pregnancy.^9^ Detailed quantitative and qualitative feedback in the Attitudes to Postnatal Instead of Normally-Timed Cervical Screening (pre-PINCS) acceptability study^15^ (unpublished data) alongside the previous QI project focus groups told us that new parents have many competing priorities and often struggle to address their own health needs.

National Institute for Health and Care Excellence guidelines recommend a 6-week postnatal check for mothers and babies, which is attended by 78% of eligible people.^16 17^ This appointment provides an opportunity for healthcare professionals to discuss multiple topics: infant feeding, lifestyle advice, contraception and health promotion, including discussion of cervical screening.^17 18^ New mothers and primary care staff told us that offering to combine this visit with postnatal cervical screening would remove a significant barrier, particularly as ‘just putting it off’ was the most common reason for younger women being out-of-date for screening in a study by Jo’s Cervical Cancer Trust.^19^

UK national guidance currently advises waiting 12 weeks after childbirth for a routine cervical screening test if it was due in pregnancy.^20^ This recommendation is based on one comparison of conventional cytology with Papanicolaou smear testing at 4 weeks versus 6 weeks versus 8 weeks postnatal in just 55 participants.^21^ There were increased inflammatory changes in Papanicolaou smears taken earlier, leading to more false-positive, low-grade results. However, this predates hrHPV primary testing (or triage) and liquid-based cytology (LBC), which dramatically improve the ability to test even inflammatory samples and those contaminated by blood and lochia.

An Irish observational study, including 556 postnatal women, reported no difference in inadequate cervical sample rates when the cervical sample was taken at 6 weeks postnatal using LBC compared with a non-pregnant gynaecological population consisting of 1429 women.^22^ Using LBC appears to negate the previously held belief that postnatal cervical samples should be delayed until 12 weeks postnatal. HPV testing was not conducted in this study, and there have been no studies directly comparing LBC cervical screening samples at different postnatal time points in a diagnostic test accuracy (DTA) context. Furthermore, hrHPV infection rates are similar during and outside of pregnancy, although these studies performed hrHPV tests at varying postnatal intervals, ranging from 45 days^23^ to 6 months,^24^ and used vaginal swabs rather than clinician-collected LBC samples. This variation limits the applicability of these findings to current UK practice. The current recommendations to delay cervical screening until 12 weeks postpartum are therefore based on long-held perceived wisdom, rather than sound evidence of differences in DTA using current screening methods.

Many women struggle to undergo conventional cervical screening, especially those in higher-risk and socioeconomically disadvantaged groups.^25^ Performing hrHPV testing using self-sampling methods offers an alternative and improves screening uptake in underscreened women.^26 27^ However, previous studies have not specifically targeted postnatal women,^27 28^ whose feelings on vaginal sampling may be affected by recent birth experiences. Our project also provides an opportunity to test the feasibility and acceptability^10^ of self-sampling for hrHPV in urine samples at 6 weeks and 12 weeks postnatal, alongside conventional testing.

We have investigated the acceptability of cervical screening earlier in the postnatal period in a quantitative and qualitative attitudes study (Pre-PINCS – National Institute for Health and Care Research (NIHR) Central Portfolio Management System (CPMS) ID: 55489).^15^ Preliminary analyses suggest that over two-thirds of respondents would be willing to take part in a clinical study of 6 week clinician-taken cervical screening, and nearly 8 out of every 10 would be willing to take part in a study of self-sampling with urine samples (unpublished data).^15^ Over half of the participants agreed or strongly agreed that they would be more likely to have cervical screening if offered at the time of their postnatal check-up (unpublished data). Although this current study is set within the well-established NHS CSP, offering opportunistic cervical screening at the time of the postnatal check-up also offers significant advantages to countries without organised call-recall screening programmes.

### Aim

PINCS is a two-phase study; this protocol refers to PINCS-1, a paired-sample study design comparing postpartum cervical screening performed at 6 weeks or 12 weeks postnatal. The overall aim will be to assess the acceptability and feasibility of this study design in comparing conventional cervical screening and self-sampling at 6 weeks versus 12 weeks postnatal.

### Objectives of PINCS-1

#### Primary objective

- To evaluate the feasibility of a paired-sample study design for a future larger scale trial investigating the acceptability of cervical screening at 6 weeks postnatal and willingness to have repeat screening at 12 weeks postnatal.

#### Secondary objectives

- To evaluate the acceptability of clinician-taken cervical samples and self-collected urine samples for screening tests in those who decline, and in those who consent both at 6 weeks and 12 weeks using questionnaire data.
- To assess the quality of cervical samples from clinician-taken samples at 6 weeks postnatal through inadequacy rates.
- To determine the agreement in hrHPV status at 6 weeks and 12 weeks postnatal between clinician-taken cervical samples and self-collected urine samples.

## Methods and analysis

### Study design

PINCS-1 is a paired feasibility study^29^ to investigate the acceptability of cervical screening and urine self-sampling in postnatal women at 6 weeks and 12 weeks postnatal.

### Study setting

The primary study site will be Somerset NHS Foundation Trust. Two further study sites across South West England will collaborate in this study (Royal Devon and Exeter NHS Trust and Royal Cornwall NHS Foundation Trust), recruiting participants, completing study visits and data collection. Each site is a Gynaecological Cancer Centre. Somerset NHS Foundation Trust acts as the study sponsor. The study planned start date is April 2024 (opened August 2024). Recruitment will end when at least 100 recruited participants have attended and completed clinician-taken cervical screening at their 6-week appointment and have attended or declined to attend their 12-week appointment. If participants withdraw before the 6-week sample, further participants will be recruited, so that at least 100 participants have their 6-week samples performed. The study will end once all participants have completed follow-up, as described above, and data have been collected and analysed. In the instance of low recruitment, an earlier end point may be initiated following discussion with the Independent Trial Steering Committee (ITSC). Anticipated end date is April 2027.

### Patient and public involvement

This study was instigated following the direct request by stakeholders, when investigating methods to reduce barriers to cervical screening in recently pregnant women/people.^9^ Multiple ideas for change were generated through stakeholder groups involving new mothers, young women who had cervical cancer diagnosed shortly after pregnancy and primary care staff directly involved in both postnatal care and cervical screening. In addition to suggestions about improving education about cervical screening for midwives and pregnant women/new parents, both public and healthcare participants identified two areas to target: earlier postnatal screening potentially at the time of the postnatal GP appointment and the use of self-sampling methods.

We worked with local Maternity Voices groups, whose members included women from marginalised communities, to design study materials, questionnaires and semistructured interviews for the pre-PINCS study,^15^ which is currently undergoing analysis. Pre-PINCS was a two-phase study consisting of a questionnaire and indepth qualitative analysis of semistructured interviews. This was performed to gather information, from pregnant women and people within 5 years of their last childbirth, about the acceptability and feasibility of the PINCS studies; these results directly informed the PINCS study design and materials, with specific feedback from participants.

### Participants and recruitment

Potential participants can be identified by members of their existing clinical care team including GPs, community or hospital midwives, health visitors, practice nurses or obstetricians or will be approached if eligible by the local research teams, both antenatally and up to 6 weeks postnatal, in an inpatient or outpatient setting. Potential participants may also self-identify through the publicity literature on recruitment sites and via the social media channels of gynaecological cancer charities (eg, GO Girls, Eve Appeal) and local and national social media groups for new mothers (eg, Mumsnet). Publicity will be in the form of posters and leaflets, distributed via social media, at antenatal events and at routine appointments or shared through the electronic maternity care record. Potential participants will be given a participant information leaflet and, if interested in taking part, they will be referred to a member of the study team. A screening and eligibility questionnaire will be completed with all potential participants and, if eligible and consenting to proceed, an electronic consent form will be completed with an investigator. Participants will be informed of their right to rescind consent at any point during the study and provided with information on how to do this.

Recruitment to PINCS-1 will end when at least 100 recruited participants have attended and completed both clinician-taken cervical sample and urine self-sample at the 6-week appointment and have attended or declined to attend their 12-week appointment. If participants withdraw prior to the 6-week sample, further participants will be recruited. In the instance of low recruitment, an earlier end point may be initiated following discussion with the ITSC. The study will be performed in secondary care, to limit the number of sites required and control for variability of cervical sampling from multiple cervical screeners. This is because this study will examine the feasibility of a future large paired-sample study, comparing DTA of cervical screening at 6 weeks and 12 weeks postnatal. A different study design will be required in a further study to test the effect on uptake of cervical screening, if offered at the 6 week postnatal check-up. This further study will necessarily be conducted in primary care settings. However, we will need to confirm that this is safe and acceptable to the postnatal population before testing within the wider cervical screening programme.

#### Inclusion criteria

- Participants aged from 24.5 years (24 years and 183 days or greater on day of consent) to <65 years old.
- Female with a cervix (regardless of gender identity).
- Currently pregnant or within 6 weeks of delivery.
- Able to give informed consent.

#### Exclusion criteria

- Absence of a cervix.
- Not eligible for the NHS CSP.
- Unable to give fully informed consent.

The study is open to all those eligible for cervical screening, regardless of screening status. To understand the reasons for non-participation and to establish an uptake rate, a cohort of 100 potential participants will be approached and the acceptance rate recorded. All those who decline to participate will be given the opportunity to describe the reasons behind this. All participants who initially gave consent to the study but chose to withdraw it will be offered a short electronic questionnaire to identify any concerns and barriers to participation.

### Sample size

This study will aim to recruit at least 100 participants to the PINCS-1 study. This sample size was chosen following findings from the pre-PINCS study regarding manageable recruitment in postnatal patients as well as input from statisticians and other experienced researchers with experience in feasibility studies.^15^ This sample size will provide a SE on uptake at most 2.5% on each proportion, which we judge to be suitable for assessing acceptability and feasibility of a subsequent paired study design for accuracy. It will inform us as to how prepared women are to undergo cervical screening with a speculum examination at 6 weeks postnatal, and the feasibility of a paired-sample design using repeat testing in the same participant with clinician- and/or self-samples at both or either time points.

### Study visits

**Figure 1:**
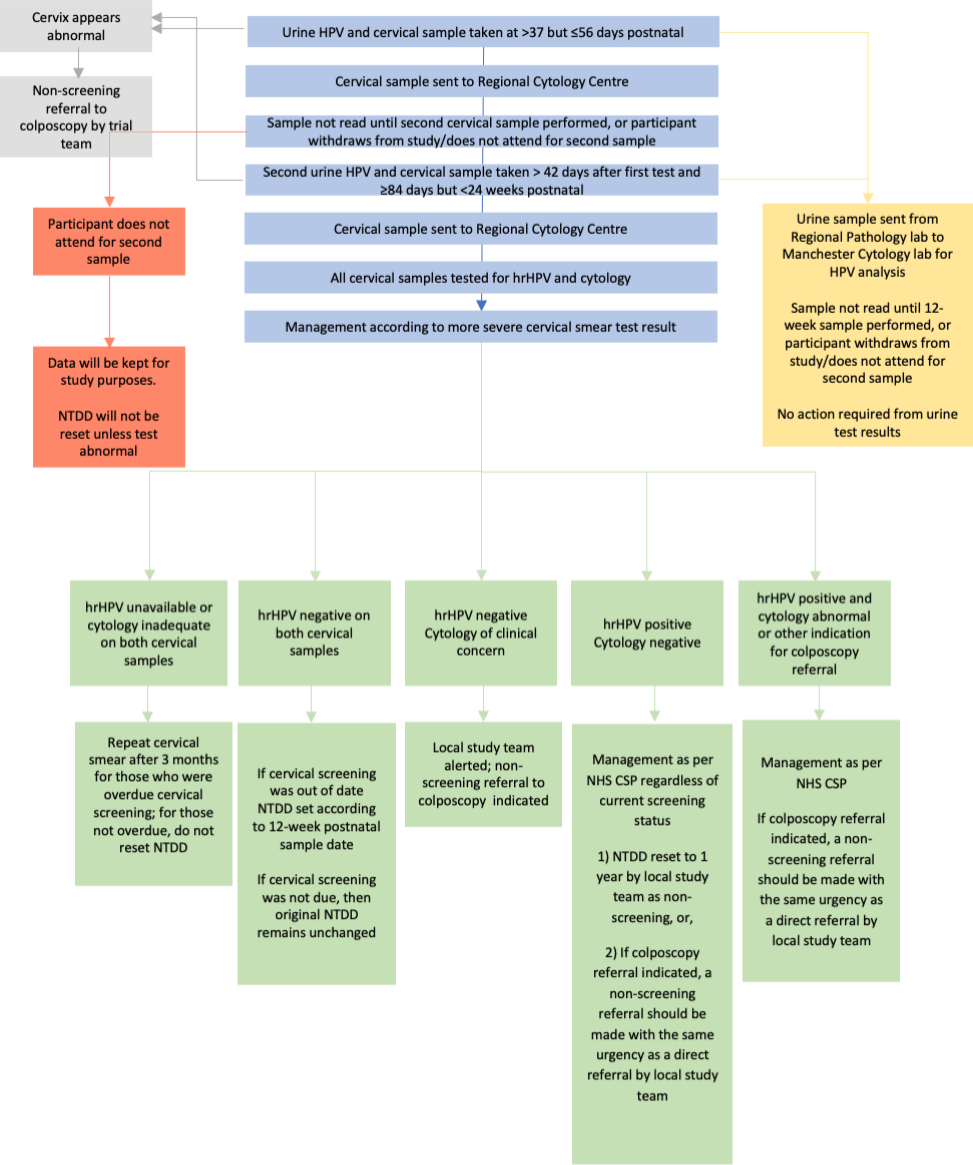
PINCS-1 participant flowchart. hrHPV, high-risk human papillomavirus; NHS CSP, National Health Service Cervical Screening Programme; NTDD, next test due date; PINCS, Postnatal Instead of Normally-Timed Cervical Screening.

The study will consist of an eligibility screening and consent appointment followed by two study visits (see figure 1). At each study visit, participants will undergo clinician-taken cervical screening samples, by an accredited clinician, using a speculum examination and Cervex brush for hrHPV testing and cytology at 6 weeks postnatal. They will also undergo hrHPV testing using first void urine samples collected with a 10 mL Colli-pee device (prior to the clinician-taken sample) at both time points, to ascertain the agreement with clinician-taken sampling and the acceptability to participants at both time points.

We will perform a patient questionnaire after sampling (web-based or paper), at both 6 weeks and 12 weeks (onlinesupplemental materials 1  2), to ascertain acceptability (concordance with protocol), feasibility (ability to recruit),^29^ patient-reported outcomes, including discomfort of testing, preferences regarding timing of screening and attitudes to introducing the option of screening at the 6 week postnatal check-up in the GP practice. This is based on a questionnaire used in a previous study, following feedback from patients and participants.

### Management of cytology and urine samples

All 6 week cervical samples will undergo initial steps in the laboratory, to allow for safe storage, and saved for processing once the 12 week sample is due. If the participant attends a 6-week sampling but subsequently withdraws from the study prior to 12 weeks, their 6-week sample will be processed and the result will be communicated to them and their GP.

All cervical samples will be processed and tested in the regional cervical cytology laboratory (North Bristol Trust) using the Hologic system. All urine samples will be tested at the cytology laboratory in Manchester using the Roche 8800 platform, as the Hologic system was not as sensitive for urine HPV analysis when compared during a previous study.^27^

Cytology samples performed following a hrHPV positive test will be dual labelled with patient identifying information and study details/study number and stored and managed in accordance with NHS CSP guidance.

Results of the cytological assessment on hrHPV negative samples, which would not ordinarily be performed as part of the NHS CSP, will not be uploaded to the NHS Cervical Screening Administration Service (CSAS), but will be recorded for the purposes of the study and acted on within the study protocol. Cytology samples from hrHPV-negative tests at 6 weeks postnatal will be destroyed at the end of the study period and not made available to CSAS for future audit.

**Figure 2:**
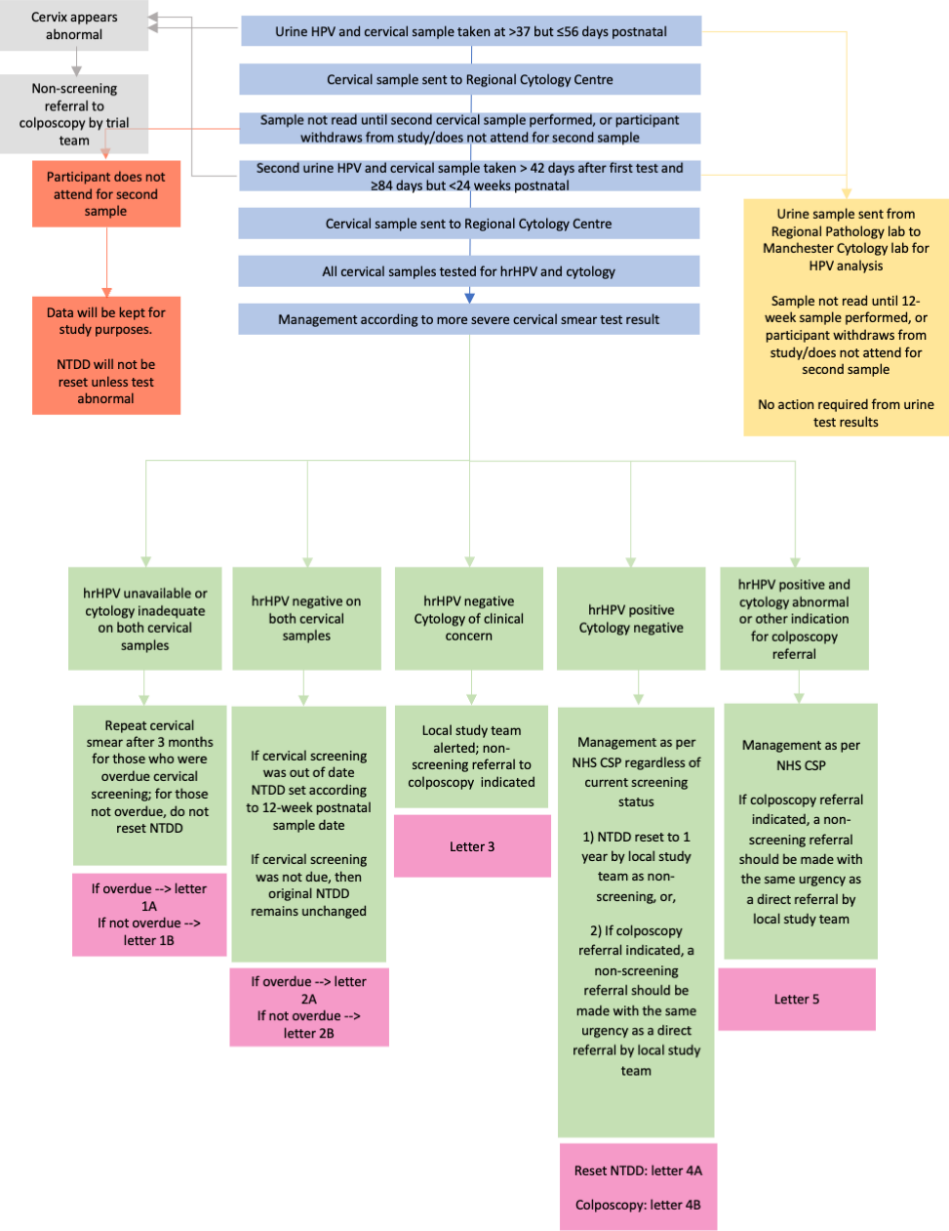
PINCS-1 study flowchart for those having samples at 6 weeks and 12 weeks. hrHPV, high risk human papillomavirus; NHS CSP, National Health Service Cervical Screening Programme; NTDD, next test due date; PINCS, Postnatal Instead of Normally-Timed Cervical Screening; PROM, patient reported outcome measures.

Management of results and further cervical screening will depend on previous cervical screening history (whether up to date at time of study or not), attendance for both samples and results of screening (see figures2  3; online supplemental material 3). The sample that demonstrates the higher-grade abnormality will determine the ongoing pathway, according to NHS CSP management guidelines. Participants will be contacted with results and management plan, questions about further management answered and asked about any adverse events, as well as being encouraged to self-report adverse events to the study team.

**Figure 3:**
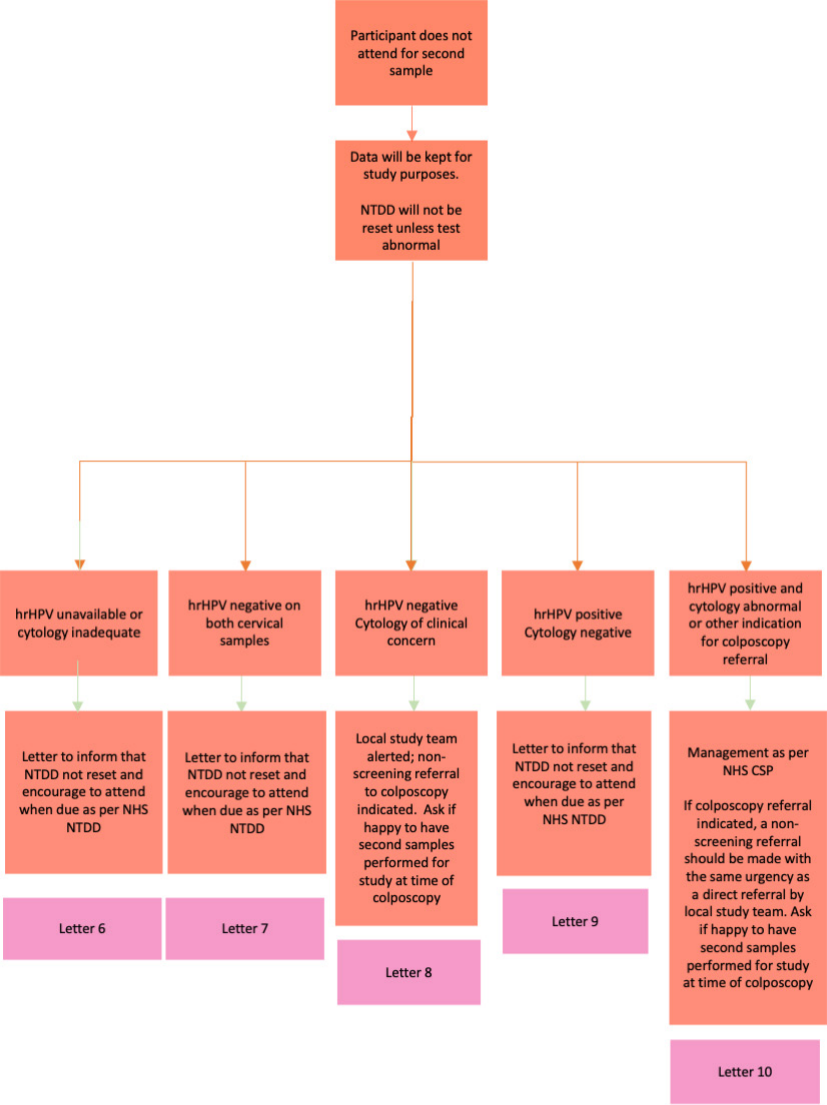
PINCS-1 study flowchart for those having samples at 6 weeks who do not attend for their 12-week sample. hrHPV, high risk human papillomavirus; NHS CSP, National Health Service Cervical Screening Programme; NTDD, next test due date; PINCS, Postnatal Instead of Normally-Timed Cervical Screening.

Urine samples will be labelled with the study details and study ID number and will be destroyed after testing and communication of results with the study team; participants will not be informed of their urine sample results.

### Data collection

Each participant will be assigned a unique study ID following consent to participate. All trial data will be uploaded to the secure web application for managing data, REDCap, which will host the electronic case report form. The study coordinators will be responsible for analysing and monitoring the data from all sites and thus will have full access to the inputted information, and local investigators will be able to access the data from their site only. Participants’ electronic notes and cervical screening records will be accessed up to 1 year after recruitment to gather data on attendance to follow-up, subsequent cervical screening results and any colposcopy assessments.

### Statistical analysis

Full details of the statistical analysis will be described in a statistical analysis plan that will be written and finalised before data lock. The primary acceptability outcomes are binary variables; the number of participants attending at 6 weeks of those who consent and the number attending at both 6 weeks and 12 weeks. We will estimate 95% CIs for each using Wilson’s method. The primary feasibility outcome is the recruitment rate in the substudy of 100 consecutive potential participants. We will compare pain scores on a 10-point scale of testing at 6 weeks and 12 weeks, using paired sample analysis and other patient-reported outcome measures. We will compare inadequacy rates of cytology samples at 6 weeks and 12 weeks. We will use 2×2 tables to analyse sensitivity and specificity of: combination HPV testing and cytology of LBC samples at 6 weeks and 12 weeks; HPV testing of LBC samples versus urine samples at both 6 weeks and 12 weeks.

## Discussion

Enhancing cervical screening uptake is a healthcare priority, as adequate screening rates lead to reduced incidences of precancerous and cancerous changes in the cervix.^4 5^ There is a clear need for research in methods to improve attendance of cervical screening in younger women due to a lack of proven strategies in the current literature.^30^ Pregnancy provides several points of contact to engage patients in health promotion through the increased access to healthcare and provides a valuable opportunity to educate and organise cervical screening, especially in ‘hardly reached’ groups.^8 9 31^ Offering opportunistic self-sampling in a healthcare setting during a pre-existing appointment with vaginal swabs to non-attenders achieved uptake rates of 55.9% in a recent study, compared with only 12.9% of those sent test kits via direct mail.^12^ They found that urine self-sampling was preferred to vaginal sampling (41.9% vs 15.4%), especially among women from ethnic minorities.^11^ From our preliminary attitudes to self-sampling data, this is likely to be even more pertinent to the postnatal cohort. However, this work also highlighted that the idea of self-sampling is not preferable to all. Women have identified making and attending appointments as a significant barrier to screening, and therefore it is essential to minimise process-based restrictions that limit accessibility to screening services.^14 32^ Combining screening with postnatal check-ups offers a golden opportunity to inform women, promote self-care and provide low-effort access to screening. This may require increased flexibility of primary care appointments, unless self-sampling is accurate enough to allow this as an alternative, and support a redirection to focus of postnatal care on maternal healthcare needs, not just those of their babies.

We outline the protocol for a study evaluating the feasibility and acceptability of cervical screening using pair-wise sampling of clinician-taken cervical screening tests and self-sampling with urine samples at 6 weeks and 12 weeks postnatal. Uptake to the study and acceptability of LBC screening at 6 weeks in the consented study sample will inform whether progression to a definitive trial is justified. We will conduct subgroup analyses of uptake based on screening status to determine the feasibility of applying these criteria for the definitive study. To maximise participation in PINCS-1, we will invite women to join regardless of their screening status at the end of pregnancy, since the aim of a subsequent paired-sample study would be to test the DTA of earlier postnatal sampling, not its effect on uptake.

Overall, through the PINCS-1 study and another study (PINCS-2—to test the feasibility of individual randomisation to 6-week vs 12-week study design), we anticipate establishing the level of acceptability and feasibility to inform the design of two further studies and how best to take these forward. First, a DTA study to determine the accuracy of screening for hrHPV and cytological abnormalities at 6 weeks postnatal will be conducted. This will compare the inadequacy rates, sensitivity and specificity of cervical screening at 6 weeks versus 12 weeks postnatal, informing whether offering earlier postnatal screening is accurate. Provisional power calculations, based on inadequacy rates, estimated requiring over 1000 participants for a formal DTA of cervical screening at 6-week postnatal, hence why this feasibility study is required before embarking on such a significant undertaking. Data from PINCS-1 will inform this study design and size for adequate power.

Second, a randomised control trial (RCT) to examine the effect of earlier postnatal screening on screening uptake rates, as well as the longer-term clinical outcomes, such as rates of high-grade cervical intraepithelial neoplasia at subsequent screening tests, will be conducted. Our proposed feasibility studies will determine whether, in this future RCT, it is reasonable and cost-effective to randomise individual participants to screening at 6 weeks or 12 weeks. If this design is not feasible, a different design will be needed. For example, randomisation without prior consent, such as through applying for a CAG-251 exemption or a pragmatic cluster-randomised design, such as that employed with YouScreen.^12^

Self-administered vaginal swabs and urine samples for hrHPV testing are under evaluation.^12 27 33^ However, this research will provide crucial insights into postnatal individuals’ experiences with and preferences for different self-sampling methods. These data will help determine the appropriate sample sizes needed to evaluate the accuracy and safety of these self-sampling techniques in future studies involving postnatal cohorts, as well as influencing future changes to the NHS CSP.

## Ethics and dissemination

### Ethics

Ethical approval for PINCS-1 was granted by the Stanmore Research Ethics Committee for this study (IRAS project ID:321696; REC reference:24/LO/0206), was adopted by the NIHR Clinical Research Network Portfolio (CPMS ID 60494) and is registered on the International Standard Randomised Controlled Trial Number (ISRCTN) registry (ISRCTN10071810; https://doi.org/10.1186/ISRCTN10071810).

### Publication and dissemination plan

Study results will be published as a PhD thesis and in high-impact peer-reviewed papers, as well as presentations at national and international meetings. They will also be presented to members of Maternity Voice Groups, gynaecological oncological charities, Mumsnet and local maternity social media sites. Any data arising from this study will be published and presented in an open-access peer-review journal. The manuscript will be deposited with the University of Exeter, according to the University of Exeter’s policies and data sharing policies.

### Individual participant data sharing statement

To ensure participant anonymity is safeguarded and subject to any reasonable and necessary delay, pseudonymised research data will be securely archived to a repository following publication of the results where they will be stored for 10 years, as per the Sponsor’s policy. These data may be used in future research, here or abroad, and shared, subject to reasonable requests, approved by the sponsor, host institution and the regulatory authorities.

### Secondary identifying numbers

CPMS 60494, MR/X030776/1, IRAS 321696.

## Supplementary material

10.1136/bmjopen-2024-092701 online supplemental material 1

10.1136/bmjopen-2024-092701 online supplemental material 2

10.1136/bmjopen-2024-092701 online supplemental material 3
