# Supplementary material for: Postnatal instead of normally-timed cervical screening (PINCS-1): a protocol for a feasibility study of paired-sample cervical screening and urine self-sampling at 6 weeks and 12 weeks postnatal in the UK
Source: BMJ Open. 2025 May 30;15(5):e092701. doi: 10.1136/bmjopen-2024-092701 (PMC12128473; doi:10.1136/bmjopen-2024-092701)
Supplement: online supplemental material 1 [file bmjopen-15-5-s001.docx]

**Before completing this questionnaire, please make sure you have read the information sheet.**

**By completing this questionnaire, you consent to take part in the study.**

**To complete the questionnaire please circle the answer most applicable to you in each question, tick the correct box or write in the space provided.**

**Knowledge about HPV**

**Before we asked you to take part in this study had you heard of HPV (Human papillomavirus)?**

Yes No Unsure

**About the tests today**

**Which words describe how you felt about having a clinician take a CERVICAL sample?** **(Please tick ALL that apply)**

Uncomfortable It was easy Embarrassed Private Reliable Convenient

Comfortable Invasive Unreliable Too soon Reassuring Overwhelming

**On a scale of 0 to 100 how uncomfortable was having the cervical sample taken today**

**(0 not at all; 100 extremely painful)? ……………………………………..**


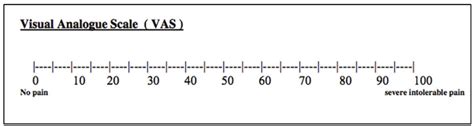


**How much do you agree with these statements? Please circle the appropriate number for each statement.**

|  | **Strongly disagree** | **Somewhat disagree** | **No opinion** | **Somewhat agree** | **Strongly agree** |
| --- | --- | --- | --- | --- | --- |
| *I felt discomfort whilst having a cervical sample* | **1** | **2** | **3** | **4** | **5** |
| *It felt unpleasant during the cervical sample* | **1** | **2** | **3** | **4** | **5** |
| *I felt embarrassed during the cervical sample* | **1** | **2** | **3** | **4** | **5** |
| *I felt anxious during the cervical sample* | **1** | **2** | **3** | **4** | **5** |
| *I felt reassured by the examination* | **1** | **2** | **3** | **4** | **5** |
| *I am worried the clinician has not collected the cervical sample correctly* | **1** | **2** | **3** | **4** | **5** |
| *I am worried how accurate the result from the cervical sample is at 6-weeks after I’ve given birth.* | **1** | **2** | **3** | **4** | **5** |
| *I would prefer a clinician to take my sample for cervical screening more than 12 weeks after giving birth.* | **1** | **2** | **3** | **4** | **5** |
| *I would be happy to have a cervical sample taken 6-weeks after giving birth, at the same time as a routine 6-week postnatal check-up* | **1** | **2** | **3** | **4** | **5** |
| *I would be happy to have a cervical sample taken 6-weeks after giving birth, but NOT at the same time as a routine 6-week postnatal check-up* | **1** | **2** | **3** | **4** | **5** |
| *In the future, I would rather delay my cervical screening to more than 12 weeks after giving birth.* | **1** | **2** | **3** | **4** | **5** |
| *If my cervical screening were due, I would be more likely to have it done, if it were offered at the same visit as the routine 6-week check up* | **1** | **2** | **3** | **4** | **5** |

**Postnatal Instead of Normally-timed Cervical Screening-1 (PINCS-1)**

**Which words describe how you felt about providing a URINE sample? (Please tick ALL that apply)**

Uncomfortable It was easy Embarrassed Private Reliable Convenient

Comfortable Invasive Unreliable Too soon Reassuring Overwhelming

**How much do you agree with these statements about URINE samples? Please circle the appropriate number for each statement.**

|  | **Strongly disagree** | **Somewhat disagree** | **No opinion** | **Somewhat agree** | **Strongly agree** |
| --- | --- | --- | --- | --- | --- |
| *I felt confident collecting a urine sample for cervical screening.* | **1** | **2** | **3** | **4** | **5** |
| *I felt discomfort whilst collecting the urine sample* | **1** | **2** | **3** | **4** | **5** |
| *It felt unpleasant collecting the urine sample* | **1** | **2** | **3** | **4** | **5** |
| *I felt embarrassed collecting the urine sample* | **1** | **2** | **3** | **4** | **5** |
| *I felt anxious collecting the urine sample* | **1** | **2** | **3** | **4** | **5** |
| *I am worried I have not collected the urine sample correctly* | **1** | **2** | **3** | **4** | **5** |
| *I am worried how accurate the urine sample is.* | **1** | **2** | **3** | **4** | **5** |
| *I would prefer a clinician to take my sample for cervical screening than provide a sample myself.* | **1** | **2** | **3** | **4** | **5** |
| *A cervical sample taken by a clinician is more reliable.* | **1** | **2** | **3** | **4** | **5** |
| *I would prefer to take my own urine sample for cervical screening* | **1** | **2** | **3** | **4** | **5** |
| *I would prefer to take my own vaginal swab sample for cervical screening* | **1** | **2** | **3** | **4** | **5** |
| *I felt I understood the instructions that were given to me.* | **1** | **2** | **3** | **4** | **5** |
| *I found it easy to collect a urine sample using the container provided.* | **1** | **2** | **3** | **4** | **5** |
| *I would prefer to take the urine sample more than 12 weeks after giving birth.* | **1** | **2** | **3** | **4** | **5** |
| *I would be happy to have a urine sample taken 6-weeks after giving birth, at the same time as a routine postnatal check-up* | **1** | **2** | **3** | **4** | **5** |
| *I would be happy to have a urine sample taken 6-weeks after giving birth, but NOT at the same time as a routine postnatal check-up* | **1** | **2** | **3** | **4** | **5** |
| *If my cervical screening were due, I would be more likely to have it done, if it were offered as a urine sample at the same visit as the routine 6-week postnatal check up* | **1** | **2** | **3** | **4** | **5** |

In the future, the NHS Cervical Screening Programme might offer a choice between using a **URINE or VAGINAL** self test

**Cervical screening in the future**

**In the future, would you prefer to do a self-test or have a healthcare professional do the test?**

🞏 Prefer a self-test with a urine sample

🞏 Prefer a self-test with a vaginal swab

🞏 Prefer a healthcare professional to do the test

🞏 No preference

**If you were offered a self-test in the future, would you rather get it…**

🞏 In the post

🞏 In person at the GP surgery

🞏 No preference

at home, or going for a cervical screening appointment with a nurse or doctor (we call this ‘**clinician**

**testing**’). Thinking about this, please tell us how much you agree or disagree with the following statements. (Please circle the appropriate number for each statement)

|  | **Strongly disagree** | **Somewhat disagree** | **No opinion** | **Somewhat agree** | **Strongly agree** |
| --- | --- | --- | --- | --- | --- |
| I would like to be offered a choice between self-testing and clinician testing for cervical screening | **1** | **2** | **3** | **4** | **5** |
| I would feel worried about being offered a choice between self-testing and clinician testing for my cervical screening | **1** | **2** | **3** | **4** | **5** |
| I would not want to be offered a choice between self-testing and clinician testing for my cervical screening | **1** | **2** | **3** | **4** | **5** |
| If I was given the choice between self-testing and clinician testing for cervical screening, I would assume it was a way of saving the NHS money | **1** | **2** | **3** | **4** | **5** |
| Being offered a choice between self-testing and clinician testing for cervical screening makes sense to me | **1** | **2** | **3** | **4** | **5** |
| I would find it difficult to choose between self-testing and clinician testing for cervical screening | **1** | **2** | **3** | **4** | **5** |
| Offering a choice between self-testing and clinician testing would improve cervical screening for me | **1** | **2** | **3** | **4** | **5** |
| I would prefer to have a recommendation to do either self-testing or clinician testing rather than having to make a choice myself | **1** | **2** | **3** | **4** | **5** |
| If I tested positive for HPV virus in the urine, I would be more inclined to go for a smear test | **1** | **2** | **3** | **4** | **5** |

**Cervical screening in the past**

**Is this the first time you have been invited for cervical screening?**

Yes No Unsure

**Have you always attended for cervical screening when invited in the past?**

Yes No Unsure Not applicable

**Have you ever delayed attending for cervical screening**?

Yes No Unsure

**Have any of the following put you off cervical screening?**

Embarrassment about having the test 🞏 Yes 🞏 No 🞏 Not sure

Worry about pain or discomfort 🞏 Yes 🞏 No 🞏 Not sure

Difficulty making a convenient appointment 🞏 Yes 🞏 No 🞏 Not sure

Difficulty taking time off work 🞏 Yes 🞏 No 🞏 Not sure

Just not getting round to it 🞏 Yes 🞏 No 🞏 Not sure

Being too busy to go for screening 🞏 Yes 🞏 No 🞏 Not sure

Not feeling at risk of cervical cancer 🞏 Yes 🞏 No 🞏 Not sure

A previous bad experience of screening 🞏 Yes 🞏 No 🞏 Not sure

Not having any symptoms 🞏 Yes 🞏 No 🞏 Not sure

Don’t like getting undressed in public 🞏 Yes 🞏 No 🞏 Not sure

Fear of what the test might find 🞏 Yes 🞏 No 🞏 Not sure

Having other health problems 🞏 Yes 🞏 No 🞏 Not sure

**Has anything else put you off?** ­­­­­­­­­­­­­­­­­­­­­­­­­___________________________________________________________________________________

__________________________________________________________________________________

**About you**

**Which of the following best describes your ethnicity? Please tick your answer below**

| **White**  British  Irish  Gypsy or Irish Traveller  White Other, please describe: | **Multiple ethnic groups**  ☐White and Black Caribbean  ☐White and Black African  ☐White and Asian  ☐Multiple other, please describe: | **Asian/ Asian British**  ☐Indian  ☐Pakistani  ☐Bangladeshi  ☐Chinese  Asian other, please describe: | **Black/African/Caribbean/ Black British**  ☐African  ☐Caribbean  ☐Black/African/Caribbean other, please describe: |
| --- | --- | --- | --- |
| **Other ethnic group**  ☐Arab ☐Other, please describe: | | ☐Prefer not to say | |

**How would you best describe your employment status? Please tick your answer below**

Employed Unemployed Student Full time parent/carer Retired

**Which of these qualifications do you have? Please tick all that apply**

Apprenticeship

GCE O-level/GCSE or equivalent

NVQ or equivalent (including BTEC general/national, OND or ONC, City and Guilds Craft)

AS, A-level or equivalent

Degree or above (including HND or HNC, NVQ level 4 or above, teaching and nurse degree)

Postgraduate e.g. second qualification such as masters, PGCERT, PhD

None

Other (please specify) …………………………………………………………………………………………….

**Which of the following describes how you think of yourself? Please tick your answer below**

Woman Man (including trans man) Non-binary

Other (please specify)………………………………………

Prefer not to say

**Is your gender the same as the gender you were given at birth? Please tick your answer below**

Yes No  Prefer not to say

**Which of the following describes how you think of yourself? Please tick your answer below**

Heterosexual or Straight Lesbian or Gay Bisexual

Other sexual orientation not listed (please specify)……………………………………………………..

Prefer not to say

**Which of the following describes how you think of yourself? Please tick your answer below**

I do not consider myself to be disabled Physical disability (including sensory impairment) Learning disability (including development disorders) Another experience of disability

Prefer not to say
