# Supplementary material for: Postnatal instead of normally-timed cervical screening (PINCS-1): a protocol for a feasibility study of paired-sample cervical screening and urine self-sampling at 6 weeks and 12 weeks postnatal in the UK
Source: BMJ Open. 2025 May 30;15(5):e092701. doi: 10.1136/bmjopen-2024-092701 (PMC12128473; doi:10.1136/bmjopen-2024-092701)
Supplement: online supplemental material 2 [file bmjopen-15-5-s002.docx]

**Before completing this questionnaire, please make sure you have read the information sheet.**

**By completing this questionnaire, you consent to take part in the study.**

**To complete the questionnaire please circle the answer most applicable to you in each question, tick the correct box or write in the space provided.**

**Postnatal Instead of Normally-timed Cervical Screening-1 (PINCS-1)**

**About the tests today**

**Which words describe how you felt about having a clinician take a CERVICAL sample?** **(Please tick ALL that apply)**

Uncomfortable It was easy Embarrassed Private Reliable Convenient

Comfortable Invasive Unreliable Too soon Reassuring Overwhelming

**On a scale of 0 to 100 how uncomfortable was having the cervical sample taken today**

**(0 not at all; 100 extremely painful)? ……………………………………..**


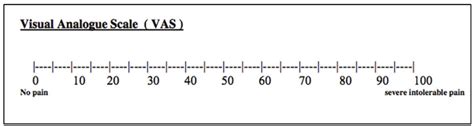


**How much do you agree with these statements? Please circle the appropriate number for each statement.**

|  | **Strongly disagree** | **Somewhat disagree** | **No opinion** | **Somewhat agree** | **Strongly agree** |
| --- | --- | --- | --- | --- | --- |
| *I felt discomfort whilst having a cervical sample* | **1** | **2** | **3** | **4** | **5** |
| *It felt unpleasant during the cervical sample* | **1** | **2** | **3** | **4** | **5** |
| *I felt embarrassed during the cervical sample* | **1** | **2** | **3** | **4** | **5** |
| *I felt anxious during the cervical sample* | **1** | **2** | **3** | **4** | **5** |
| *I felt reassured by the examination* | **1** | **2** | **3** | **4** | **5** |
| *I am worried the clinician has not collected the cervical sample correctly* | **1** | **2** | **3** | **4** | **5** |
| *I am worried how accurate the result from the cervical sample is at 6-weeks after I’ve given birth.* | **1** | **2** | **3** | **4** | **5** |
| *I would prefer a clinician to take my sample for cervical screening more than 12 weeks after giving birth.* | **1** | **2** | **3** | **4** | **5** |
| *I would be happy to have a cervical sample taken 6-weeks after giving birth, at the same time as a routine 6-week postnatal check-up* | **1** | **2** | **3** | **4** | **5** |
| *I would be happy to have a cervical sample taken 6-weeks after giving birth, but NOT at the same time as a routine 6-week postnatal check-up* | **1** | **2** | **3** | **4** | **5** |
| *In the future, I would rather delay my cervical screening to more than 12 weeks after giving birth.* | **1** | **2** | **3** | **4** | **5** |
| *If my cervical screening were due, I would be more likely to have it done, if it were offered at the same visit as the routine 6-week check up* | **1** | **2** | **3** | **4** | **5** |

**Which words describe how you felt about providing a URINE sample? (Please tick ALL that apply)**

Uncomfortable It was easy Embarrassed Private Reliable Convenient

Comfortable Invasive Unreliable Too soon Reassuring Overwhelming

**How much do you agree with these statements about URINE samples? Please circle the appropriate number for each statement.**

|  | **Strongly disagree** | **Somewhat disagree** | **No opinion** | **Somewhat agree** | **Strongly agree** |
| --- | --- | --- | --- | --- | --- |
| *I felt confident collecting a urine sample for cervical screening.* | **1** | **2** | **3** | **4** | **5** |
| *I felt discomfort whilst collecting the urine sample* | **1** | **2** | **3** | **4** | **5** |
| *It felt unpleasant collecting the urine sample* | **1** | **2** | **3** | **4** | **5** |
| *I felt embarrassed collecting the urine sample* | **1** | **2** | **3** | **4** | **5** |
| *I felt anxious collecting the urine sample* | **1** | **2** | **3** | **4** | **5** |
| *I am worried I have not collected the urine sample correctly* | **1** | **2** | **3** | **4** | **5** |
| *I am worried how accurate the urine sample is.* | **1** | **2** | **3** | **4** | **5** |
| *I would prefer a clinician to take my sample for cervical screening than provide a sample myself.* | **1** | **2** | **3** | **4** | **5** |
| *A cervical sample taken by a clinician is more reliable.* | **1** | **2** | **3** | **4** | **5** |
| *I would prefer to take my own urine sample for cervical screening* | **1** | **2** | **3** | **4** | **5** |
| *I would prefer to take my own vaginal swab sample for cervical screening* | **1** | **2** | **3** | **4** | **5** |
| *I felt I understood the instructions that were given to me.* | **1** | **2** | **3** | **4** | **5** |
| *I found it easy to collect a urine sample using the container provided.* | **1** | **2** | **3** | **4** | **5** |
| *I would prefer to take the urine sample more than 12 weeks after giving birth.* | **1** | **2** | **3** | **4** | **5** |
| *I would be happy to have a urine sample taken 6-weeks after giving birth, at the same time as a routine postnatal check-up* | **1** | **2** | **3** | **4** | **5** |
| *I would be happy to have a urine sample taken 6-weeks after giving birth, but NOT at the same time as a routine postnatal check-up* | **1** | **2** | **3** | **4** | **5** |
| *If my cervical screening were due, I would be more likely to have it done, if it were offered as a urine sample at the same visit as the routine 6-week postnatal check up* | **1** | **2** | **3** | **4** | **5** |

**Cervical screening at 6 weeks versus 12 weeks**

**Have you something you would like to share with us about having cervical screening at 6 weeks postnatal rather than at a later appointment? What would be the benefits of disadvantages to having this at the same visit as the 6-week check up with the GP practice? This might be part of the same appointment or as a double appointment before/after with a practice nurse.**

_________________________________________________________________________________

_________________________________________________________________________________

_________________________________________________________________________________

_________________________________________________________________________________

_________________________________________________________________________________

_________________________________________________________________________________

**Cervical screening in the future**

**In the future, if you needed a cervical screening test, would you prefer to have the at the same time as a visit to your GP practice for a routine 6-week postnatal check-up?**

🞏 Prefer at 6-week postnatal check up

🞏 Prefer at a separate appointment more than 12 weeks after giving birth

🞏 No preference

🞏 other – please state ……………………………………………………………………………………………………………………….

**In the future, if offered a self-sampling urine test, when would prefer this to be offered?**

🞏 Prefer at 6-week postnatal check up

🞏 Prefer a separate appointment more than 12 weeks after giving birth

🞏 No preference
