# Supplementary material for: Postnatal instead of normally-timed cervical screening (PINCS-1): a protocol for a feasibility study of paired-sample cervical screening and urine self-sampling at 6 weeks and 12 weeks postnatal in the UK
Source: BMJ Open. 2025 May 30;15(5):e092701. doi: 10.1136/bmjopen-2024-092701 (PMC12128473; doi:10.1136/bmjopen-2024-092701)
Supplement: online supplemental material 3 [file bmjopen-15-5-s003.docx]

Letters:

[Letter 1A: 1](#_Toc163743907)

[Letter 1B 1](#_Toc163743908)

[Letter 2A 2](#_Toc163743909)

[Letter 2B 2](#_Toc163743910)

[Letter 3 3](#_Toc163743911)

[Letter 4A 3](#_Toc163743912)

[Letter 4B 4](#_Toc163743913)

[Letter 4C 4](#_Toc163743914)

[Letter 5 4](#_Toc163743915)

[Letter 6 4](#_Toc163743916)

[Letter 7 5](#_Toc163743917)

[Letter 8 5](#_Toc163743918)

[Letter 9 6](#_Toc163743919)

[Letter 10 6](#_Toc163743920)

## Letter 1A:

Dear _____

I am writing to you regarding your cervical screening results from the PINCS-1 study. Thank you for your participation in our study.

Both of your cervical screening results were inadequate for testing for the human papillomavirus (HPV) and the cell assessment (cytology).

As your cervical screening test is due, as per the normal NHS cervical screening program protocol, we recommend that you have a repeat cervical screening test at your GP practice in three months’ time. We have also sent this letter to your GP into this letter to inform them of this.

If you have any symptoms such as bleeding between periods, after sex or after the menopause, or unusual vaginal discharge, please speak with your GP as soon as possible.

If you have any questions or concerns, or you have experienced any complications or adverse events as a result of the samples taken in the study, please contact your local study team on ____

Yours sincerely,

## Letter 1B

Dear _____

I am writing to you regarding your cervical screening results from the PINCS-1 study. Thank you for your participation in our study.

Both of your cervical screening results were inadequate for testing for the human papillomavirus (HPV) and the cell assessment (cytology).

As your cervical screening test was not due at the time of the study, no action needs to be taken at this time. We recommend you attend for your cervical screening test when it is next due, which you will be informed of by the NHS cervical screening program.

If you have any symptoms such as bleeding between periods, after sex or after the menopause, or unusual vaginal discharge, please speak with your GP as soon as possible.

If you have any questions or concerns, or you have experienced any complications or adverse events as a result of the samples taken in the study, please contact your local study team on ____

Yours sincerely,

## Letter 2A

Dear _____

I am writing to you regarding your cervical screening results from the PINCS-1 study. Thank you for your participation in our study.

Both of your cervical screening results were negative for HPV (human papillomavirus) and there were no abnormal cells found (negative cytology). This means your risk of cervical cancer is very low.

As your cervical screening test was due, the date for your next test will be reset based upon the date of the second sample that you had as part of the study. You will receive a reminder letter from the NHS cervical screening program closer to the time. If you have not received a letter in three years' time, please contact your GP.

If you have any symptoms such as bleeding between periods, after sex or after the menopause, or unusual vaginal discharge, please speak with your GP as soon as possible.

If you have any questions or concerns, or you have experienced any complications or adverse events as a result of the samples taken in the study, please contact your local study team on ____

Yours sincerely,

## Letter 2B

Dear _____

I am writing to you regarding your cervical screening results from the PINCS-1 study. Thank you for your participation in our study.

Both of your cervical screening results were negative for HPV (human papillomavirus) and there were no abnormal cells found (negative cytology). This means your risk of cervical cancer is very low.

As your cervical screening test was not due, the date for your next cervical screening will not be changed. You will receive a reminder letter from the NHS cervical screening program when it is due. We recommend you attend as usual, irrespective of your study sample results.

If you have any symptoms such as bleeding between periods, after sex or after the menopause, or unusual vaginal discharge, please speak with your GP as soon as possible.

If you have any questions or concerns, or you have experienced any complications or adverse events as a result of the samples taken in the study, please contact your local study team on ____

Yours sincerely,

## Letter 3

Dear _____

I am writing to you regarding your cervical screening results from the PINCS-1 study. Thank you for your participation in our study.

Both of your cervical screening results were negative for HPV (human papillomavirus) but at least one sample showed some cells of concern (abnormal cytology). Outside of the study, if a cervical screening test was negative for HPV, we would not have gone on to examine the cells (cytology) and your sample would have been recorded as normal, so these changes are likely to not represent anything significant.

Cervical screening result: ____

However, as a precaution, we recommend a colposcopy examination to look closely at your cervix to see if there is anything that might need a biopsy and/or treatment.

You will receive an appointment for a colposcopy appointment, with a leaflet explaining the procedure.

If you have any questions or concerns, or you have experienced any complications or adverse events as a result of the samples taken in the study, please contact your local study team on ____

Yours sincerely,

## Letter 4A

Dear _____

I am writing to you regarding your cervical screening results from the PINCS-1 study. Thank you for your participation in our study.

At least one of your samples was positive for HPV (human papillomavirus). When this result is found, the sample is tested to see if there were any abnormal cells (cytology), this was reassuring (negative) on both of your samples.

As your previous results were normal, the date for your next cervical screening test will be reset to one year’s time. You will receive a reminder letter from the NHS cervical screening program when it is due. It is important to attend for this screening.

If you have any questions or concerns, or you have experienced any complications or adverse events as a result of the samples taken in the study, please contact your local study team on ____

Yours sincerely,

## Letter 4B

Dear _____

I am writing to you regarding your cervical screening results from the PINCS-1 study. Thank you for your participation in our study.

At least one of your samples was positive for HPV (human papillomavirus). When this result is found, the sample is tested to see if there were any abnormal cells (cytology), this was reassuring (negative) on both of your samples.

Because your last two cervical screening tests were also positive for HPV, even though the cells look normal, we recommend a colposcopy examination to look closely at your cervix, to see if there is anything that might need a biopsy and/or treatment.

You will receive an appointment for a colposcopy appointment, with a leaflet explaining the procedure.

If you have any questions or concerns, or you have experienced any complications or adverse events as a result of the samples taken in the study, please contact your local study team on ____

Yours sincerely,

## Letter 5

Dear ____

I am writing to you regarding your cervical screening results from the PINCS-1 study. Thank you for your participation in our study.

At least one of your samples was positive for HPV (human papillomavirus). When this result is found, the sample is tested for abnormal cells (cytology) and at least one of your samples showed some cells of concern.

Cervical screening result: ____

We recommend a colposcopy examination to look closely at your cervix, to see if there is anything that might need a biopsy and/or treatment.

You will receive an appointment for a colposcopy appointment, with a leaflet explaining the procedure.

If you have any questions or concerns, or you have experienced any complications or adverse events as a result of the samples taken in the study, please contact your local study team on ____

Yours sincerely,

## Letter 6

Dear _____

I am writing to you regarding your cervical screening result from the PINCS-1 study. Thank you for your participation in our study. As you were not able to attend for the second cervical screening test in the study, this result is from the first sample.

Your cervical screening result was inadequate for testing for the human papillomavirus (HPV) and the cell assessment (cytology).

There has therefore been no change to when your cervical screening test is next due. As this test is not part of the national screening program, if your cervical screening test was due at the time of the study, we recommend you arrange an appointment with your GP to have a cervical screening test.

If your cervical screening test was not due at the time of the study, we recommend you attend when it is next due, you will receive a reminder letter from the NHS cervical screening program closer to the time.

If you have any symptoms such as bleeding between periods, after sex or after the menopause, or unusual vaginal discharge, please speak with your GP as soon as possible.

If you have any questions or concerns, or you have experienced any complications or adverse events as a result of the samples taken in the study, please contact your local study team on ____

Yours sincerely,

## Letter 7

Dear _____

I am writing to you regarding your cervical screening result from the PINCS-1 study. Thank you for your participation in our study. As you were not able to attend for the second cervical screening test in the study, this result is from the first sample.

Your cervical screening result was negative for HPV (human papillomavirus) and there were no abnormal cells found (negative cytology).

As this test is not part of the national screening program, the date for your next cervical screening will not be changed. You will receive a reminder letter from the NHS cervical screening program when it is due. We recommend you attend as usual, irrespective of your study sample results.

If you have any symptoms such as bleeding between periods, after sex or after the menopause, or unusual vaginal discharge, please speak with your GP as soon as possible.

If you have any questions or concerns, or you have experienced any complications or adverse events as a result of the samples taken in the study, please contact your local study team on ____

Yours sincerely,

## Letter 8

Dear _____

I am writing to you regarding your cervical screening result from the PINCS-1 study. Thank you for your participation in our study. As you were not able to attend for the second cervical screening test in the study, this result is from the first sample.

Your cervical screening result was negative for HPV (human papillomavirus) but at least one sample showed some cells of concern (abnormal cytology). Outside of the study, if a cervical screening test was negative for HPV, we would not have gone on to examine the cells (cytology) and your sample would have been recorded as normal, so these changes are likely to not represent anything significant.

Cervical screening result: ____

However, as a precaution, we recommend a colposcopy examination to look closely at your cervix to see if there is anything that might need a biopsy and/or treatment.

You will receive an appointment for a colposcopy appointment, with a leaflet explaining the procedure.

If you have any questions or concerns, or you have experienced any complications or adverse events as a result of the samples taken in the study, please contact your local study team on ____. Please could you also contact the study team if you would consider having the second cervical screening test for the study at the time of the colposcopy, this is not a requirement and will not affect your care.

Yours sincerely,

## Letter 9

Dear _____

I am writing to you regarding your cervical screening result from the PINCS-1 study. Thank you for your participation in our study. As you were not able to attend for the second cervical screening test in the study, this result is from the first sample.

Your sample was positive for HPV (human papillomavirus). When this result is found, the sample is tested for abnormal cells, this was negative on your sample.

As this test is not part of the national screening program, the date for your next cervical screening will not be changed. You will receive a reminder letter from the NHS cervical screening program when it is due. We recommend you attend as usual, irrespective of your study sample results.

If you have any symptoms such as bleeding between periods, after sex or after the menopause, or unusual vaginal discharge, please speak with your GP as soon as possible.

If you have any questions or concerns, or you have experienced any complications or adverse events as a result of the samples taken in the study, please contact your local study team on ____

Yours sincerely,

## Letter 10

Dear _____

I am writing to you regarding your cervical screening result from the PINCS-1 study. Thank you for your participation in our study. As you were not able to attend for the second cervical screening test in the study, this result is from the first sample.

Your sample was positive for HPV (human papillomavirus). When this result is found, the sample is tested for abnormal cells, this identified cells of concern in your sample.

Cervical screening result: ____

We recommend a colposcopy examination to look closely at your cervix, to see if there is anything that might need a biopsy and/or treatment.

You will receive an appointment for a colposcopy appointment, with a leaflet explaining the procedure.

If you have any questions or concerns, or you have experienced any complications or adverse events as a result of the samples taken in the study, please contact your local study team on ____. Please could you also contact the study team if you would consider having the second cervical screening test for the study at the time of the colposcopy, this is not a requirement and will not affect your care.

Yours sincerely,
