# Supplementary material for: Postnatal instead of normally-timed cervical screening (PINCS-1): a protocol for a feasibility study of paired-sample cervical screening and urine self-sampling at 6 weeks and 12 weeks postnatal in the UK
Source: BMJ Open. 2025 May 30;15(5):e092701. doi: 10.1136/bmjopen-2024-092701 (PMC12128473; doi:10.1136/bmjopen-2024-092701)
Supplement: Supplementary file 4 [file bmjopen-2024-092701.reviewer_comments.pdf]

PEER REVIEW HISTORY

BMJ Open publishes all reviews undertaken for accepted manuscripts. Reviewers are asked to complete a checklist review form and are provided with free text boxes to elaborate on their assessment. These free text comments are reproduced below.

ARTICLE DETAILS

Title (Provisional)

Postnatal Instead of Normally Timed Cervical Screening (PINCS-1): a protocol for a feasibility study of paired-sample cervical screening and urine self-sampling at 6- and 12-weeks postnatal in the United Kingdom

Authors

Cullimore, Victoria; Newhouse, Rebecca; Baker-Rand, Holly; Brentnall, Adam; Chu, Kim; Denton, Karin; McWilliams, Lorna; Sargent, Alex; Sundar, Sudha; Crosbie, Emma J; Morrison, Jo

---

VERSION 1 - REVIEW

**Reviewer**	**1**
**Name**	**tranberg, mette**
**Affiliation Programmes**	**Randers Reg Hosp, Department of Public Health**
**Date**	**08-Dec-2024**
**COI**	**None**

"BMJopen-2024-092701":

Thank you for the opportunity to review this interesting protocol article. The paper addresses a significant and timely issue, contributing to the ongoing debate on how to obtain high coverage among younger in cervical cancer screening programs. It is innovative to consider urine self-sampling as part of the postnatal examinations at the GPs. However, I have several suggestions to enhance clarity and strengthen the study's aim, methods, etc.

General comments: Throughout the paper: Consider using cervical cancer screening and not just cervical screening. Check to write in the past tense. Should the study be registered at clinicaltrials.gov?

TITLE

1. Consider including country information and urine self-sampling in the title.

Abstract:
- Please mention the study aim in the introduction
- Methods and analysis: please mention the age of the study participants and the timeline for the study recruitment.
- Please clarify if women are offered both clinician-collected cervical samples and urine samples at both 6 and 12 weeks postnatal? Please clarify if it is first-void urine collection.
- Consider omitting *this will inform whether women are prepared to undergo CC screening* etc as these lines are not the methods but more perspective in my opinion. Please consider omitting the description of the second phase, the RCT part, especially as the authors mention that a separate study protocol will be published. Please clearly state the study endpoints.

Ethics and dissemination
….Clinical impact of CC screening at 6 weeks postnatal, please clarify, impact of CC screening based on urine-HPV-based screening? Or what do the authors mean?

Strengths and limitations:
- The first study to …, clearly state including urine self-sampling? And have the authors checked clinicaltrials.gov for similar studies? Perhaps write to our knowledge this study is the first to

- Omit the description of the YouScreen trial as a strength of this current study or rewrite this text.
- Limitations:
  please check to write in the past tense. This study **will have** limited sample size.

Introduction:
- I like the introduction and the study rationale is well-written. However, I miss that the authors also perspective the problem statement regarding lacking screening coverage in other countries with organized CC screening programs. This will make the study relevant to scientists and clinicians from other countries.
Line 16, page 6: when referring to the YouScreen trial please state offering vaginal self-sampling.
- what is NICE, an abbreviation for?
Line 54, page 6: national screening guidelines?
p.7, line 16 hrHPV infection rates are similar, please insert in swabs to clarify
p.7, line 37, change self-testing to self-sampling, and state whether you are performing hrHPV testing in FVU or urine. I miss references on the diagnostic accuracy of urinary HPV testing vs cervical HPV testing for CIN2+/CIn3+ detection. The importance of using FVU and a standardized first-void urine collection device as shown by Gilham et al (BJOG)
p.8, include 1 in pincs. There is redundancy in lines 3-10 and the primary study objective. Please consider omitting or reformulate.

Objective:
The primary objective will be, please change to past tense.
Secondary objectives: 2) quality of clinician-collected LBC cervical samples? Please clarify, the morphological quality.

Methods and analysis:
Study design: omit to investigate etc as this is redundancy of the study objective. Just state the study design and align it with the design written in the introduction, two-phase study with paired-sample study design.
Study setting: State the number of study sites.
Participants and recruitment:
State the timeline for the study inclusion.
Sample size:
Please insert references to the feasibility study that the experienced researchers have published.

Please consider omitting the considerations regarding the sample size of the PINCS2 study especially as this will be reported in a separate study protocol paper.
Study visits:
- Please clarify that you applied co-testing with hrhPV testing and cytology for cervical scrapes and using first-void urine samples. Please state if the 10 or 20-ml ColliPee device is used.
Which hrHPV-test assay is used? Importantly, state the order of the cervical and FVU sampling, are the cervical samples always taken first followed by the FVU samples? If so, I recommend the authors to always start med the FVU collection as you risk several invalid HPV results for the FVU samples due to the risk of blood following the cervical sampling.
- Has the patient questionnaire been properly validated?
Statistical analysis:
I would assume that in a study protocol paper, you would have decided on the final statistical analysis plan.

Discussion:
The discussion is quite lengthy, please consider omitting some of the information about the upcoming RCT trial, as this will be reported elsewhere.

p .12, lines: 47-48. Also in Denmark, we have found that ethnic minority groups prefer urine over vaginal sampling. You could consider stating that the finding in the YouScreen trial also is seen in other countries including Denmark. Feel free to use the Danish reference, "Well, That Was Pretty Clever!"-Ethnic Minority Women's Views on HPV Self-Sampling Devices for Cervical Cancer Screening: Attitudes to Brushes, First-Void Urine, and Menstrual Blood Devices"

- Another important outcome in this feasibility study and/or RCT trial should be the compliance to follow-up among women who tested hrHPV positive in their self-sample. I think it is important to get some perspectives from the women about this matter and not just if they will attend self-sampling 6 weeks postnatal. If high compliance to follow-up is not obtained when the self-sampling offer provides more harm than preventive benefits. I miss this important perspective in the discussion.

When writing that urine samples for hrHPV testing are under evaluation, please consider not just the UK studies, but also the Belgian VALHUDES studies by Van Keer and colleagues.

What about the clinical management of the women, if the woman is HPV+ with HSIL cytology and referred directly for colposcopy showing CIN3+, when will it then be ok to perform the LEETZ procedure, etc? Do the authors have opinions about this matter?

**Reviewer**	**2**
**Name**	**Rohner, Eliane**
**Affiliation**	**University of Bern Institute of Social and Preventive Medicine**
**Date**	**13-Apr-2025**
**COI**	**None**

The PINCS-1 study proposed in this protocol aims to assess the feasibility of performing cervical cancer screening among women at their 6-weeks postnatal visit in the UK. The study

builds on the results of the qualitative pre-PINCS study, which have not been published yet, and the study will be followed by PINCS-2 which will assess the feasibility of randomising participants to cervical cancer screening at 6 or 12 weeks postnatal. The research topic is interesting and relevant, as postnatal visits may offer a valuable opportunity to screen women for cervical cancer who may otherwise have little contact with the health system. However, I do have a number of comments/questions on the protocol which I am listing below.

Major comments

• The authors state that PINCS-2 will have its own study protocol. Nevertheless, they describe aspects like objectives or sample size considerations for PINCS-2 in the current study protocol. It has been quite difficult for me to disentangle which aspects will be part of PINCS-1 vs PINCS-2. I suggest that the authors refer all references to PINCS-2 into an outlook section.

• Do the authors plan to follow women over time to understand whether they will return for follow up assessments and treatment if needed?

• Could the authors elaborate more on why the decided to include women irrespective of their screening status? Are they not afraid that they might end up including a substantial proportion of women who do not require screening at this point (and might not be representative of the target population)?

• Outcomes should be more clearly defined than just referring to them as binary variables.

• The authors should elaborate more on the planned statistical analysis, even if a detailed statistical analysis plan will follow later on.

• The authors mention diagnostic test accuracy (sensitivity and specificity), but what will be the definition of cervical disease? Cytological results? Histology?

Minor comments,

Abstract

• Please explain what the acronym PINCS stands for

Strengths and limitations

• "Not statistically powered to evaluate the diagnostic test accuracy" –diagnostic test accuracy is not listed as an objective of PINCS-1 anyway?

Aim

• It would be helpful to remove the reference to PINCS-2 here, as it has the potential to confuse the reader

Objectives

• "The primary objective is to evaluate a paired-sample study design" – Is the objective to evaluate the study design or to assess feasibility?

• If I understand correctly, the justification for the repeat screening at 12 weeks postnatal is to assess women's willingness to undergo two cervical cancer screenings only 6 weeks apart to examine the feasibility of a future study which is supposed to examine the agreement between screening results at 6 and 12 months postnatal? It would be helpful if this was stated more clearly.

• 3rd secondary objective: do the authors plan to assess agreement between HPV status on cervical samples at 6 and 12 weeks, and the same for urine samples? Or are they interested in the agreement between the HPV status on urine and cervical samples? Are they referring to clinician- or self-collected cervical samples here?

Methods

• Can the authors provide more information on the study setting and the study sites involved? For example, are these clinics or gynecological practices?

• How do the authors plan to handle abnormal screening results at 6 weeks? Figure 1 suggests that the 6-week sample will not be tested until the 12-week sample is obtained. Some of the information in figure 1 should be added to the text to help the reader understand the pathways better.

• Why did the authors decide to include a convenience sample rather than enrolling all eligible, consenting women who attend 6-week postnatal visits? How representative will this convenience sample be? Is this how they plan to enroll women for larger RCTs in the future? The implications of choosing a convenience sample should be discussed in the limitations section.

• "… a cohort of 100 potential participates will be approached and the acceptance rate recorded" – how will participants recruited through leaflets and information events be considered in this?

• Description of sample size calculation for PINCS-1 is rather vague.

• Sample size calculation for PINCS-2 should be removed from this protocol.

• "The study will consist of a screening and consent appointment" – I assume the authors are referring to eligibility screening, not cervical cancer screening here?

• What is meant by "cervical sample/sweep test"? Is this a self-collected vaginal sample? What will they be used for in addition to the clinician-collected samples?

• Which HPV test will be used? Where will the laboratory tests be performed?

• "We will perform a patient questionnaire after sampling" – did the authors also consider administering a health care provider questionnaire to examine whether the providers

experienced challenges incorporating cervical cancer screening into the 6 weeks postnatal appointment?

• Figures 2 and 3: What do letters 1A, 2A etc refer to?

VERSION 1 - AUTHOR RESPONSE

General comments: Throughout the paper: Consider using cervical cancer screening and not just

cervical screening.

Reviewer 1 suggested changing to cervical cancer screening. However, this is inaccurate and the NHS Cervical Screening Programme (NHS CSP) refers to screening as "Cervical Screening". (even within the name of the programme), as screening is to prevent cervical cancer rather than detect it at an early stage. We will continue with terminology as recommended by the NHS CSP and used by WHO, to avoid promoting the commonly held belief that we are looking for cervical cancers (unlike most other cancer screening programmes).

Check to write in the past tense.

This was submitted as a protocol, prior to the study opening, hence is written in future tense.

Should the study be registered at clinicaltrials.gov?

The study is registered on the International Standard Randomised Controlled Trial Number Registry (ISRCTN10071810; https://doi.org/10.1186/ISRCTN10071810) as is already stated in the paper (ethics section). ISRCTN is the UK-based international trials registry and an equivalent of clinicaltrials.gov, approved by our funder; dual registration with ClinicalTrials,gov is neither permitted, nor required.

TITLE

- Consider including country information and urine self-sampling in the title.

Thank very much for this helpful suggestion. We have edited the title in accordance with this advice.

Abstract:

- Please mention the study aim in the introduction

Thank very much for this helpful suggestion. We have edited in accordance with this advice.

- Methods and analysis: please mention the age of the study participants and the timeline for the

study recruitment.

Thank very much for this helpful suggestion. We have edited in accordance with this advice.

- Please clarify if women are offered both clinician-collected cervical samples and urine samples at

both 6 and 12 weeks postnatal? Please clarify if it is first-void urine collection.

Thank very much for these helpful suggestions. We have edited in accordance with this advice.

- Consider omitting *this will inform whether women are prepared to undergo CC screening* etc as

these lines are not the methods but more perspective in my opinion.

Thank you for this opinion. However, you may have misinterpreted the purpose and aims of what is a feasibility study; this is not per se a diagnostic test accuracy study and results will inform size and design for future studies. This is a major component of the study methods and aims, i.e. feasibility and acceptability so we will not remove from this section as aligns with the aims of the study.

Please consider omitting the description of the second phase, the RCT part, especially as the authors mention that a separate study protocol will be published.

Thank very much for these helpful suggestions. We have edited in accordance with this advice.

Please clearly state the study endpoints.

We do not understand what the reviewer means by this comment, as the study endpoints are already included, as detailed below. This is a *feasibility* study, the design and limitations of which were as advised by our methodologist, who is an expert in feasibility study design, so these are the endpoints:

"The primary objective will be to evaluate the feasibility of a paired-sample study design for a larger scale trial investigating the acceptability of cervical screening at 6-weeks postpartum and willingness to have repeat screening at 12-weeks postpartum.

The secondary objectives are:

1. To evaluate acceptability of clinician-taken cervical samples and self-collected urine samples for screening tests in those who decline, and in those who consent both at 6- and 12-weeks using questionnaire data.
2. To assess the quality of cervical samples from clinician-taken samples at 6-weeks postnatal through inadequacy rates.
3. To determine the agreement in hrHPV status at 6- and 12-weeks postnatal between clinician-taken cervical samples and self-collected urine samples."

Ethics and dissemination

....Clinical impact of CC screening at 6 weeks postnatal, please clarify, impact of CC screening based

on urine-HPV-based screening? Or what do the authors mean?

We do not understand this comment as we explain the 'clinical impact' in the subsequent clause. However, we have added: (e.g., from timing of appointments and/or offering self-sampling)

Strengths and limitations:

- The first study to ..., clearly state including urine self-sampling? And have the authors checked

clinicaltrials.gov for similar studies? Perhaps write to our knowledge this study is the first to

Thank you for this suggestion. Amended as suggested.

- Omit the description of the YouScreen trial as a strength of this current study or rewrite this

text.

Thank you for this suggestion. Amended as suggested.

- Limitations:

please check to write in the past tense. This study **will have** limited sample size.

This is a clinical trial protocol and describes what we will do. We have changed to future tense as indicated (we were a bit confused as you said past tense but used future conditional (although we note due to the significant delay with the peer review process, this trial is now well underway!)

Introduction:

- I like the introduction and the study rationale is well-written. However, I miss that the authors also

perspective the problem statement regarding lacking screening coverage in other countries with

organized CC screening programs. This will make the study relevant to scientists and clinicians from

other countries.

Thank you for your support and this helpful suggestion. We have added "Although this study is set within the well-established NHS CSP, offering opportunistic cervical screening at the time of the postnatal check-up also offers significant advantages to countries without organised call-recall screening programmes."

Line 16, page 6: when referring to the YouScreen trial please state offering vaginal self-sampling.

Thank you – we have added this clarification

- what is NICE, an abbreviation for?

Thank you. We have added the explanation for the abbreviation.

Line 54, page 6: national screening guidelines?

"Guidance" is correct (i.e the guidance within the guidelines)

p.7, line 16 hrHPV infection rates are similar, please insert in swabs to clarify

Thank you. We have added vaginal to the sentence that was here for clarity "and used **vaginal** swabs rather than clinician-collected LBC samples."

p.7, line 37, change self-testing to self-sampling, and state whether you are performing hrHPV testing

in FVU or urine.

Thank you we have made clarifications

I miss references on the diagnostic accuracy of urinary HPV testing vs cervical HPV testing for CIN2+/CIn3+ detection. The importance of using FVU and a standardized first-void urinecollection device as shown by Gilham et al (BJOG).

We have included in reference to urine HPV DTA studies in "Davies et al" which is in BJOG and includes Gilham as an author – is this what you meant? We note comments from the Editor re no need to include further references.

p.8, include 1 in pincs.

We have reworded for clarification "PINCS is a two-phase study, this protocol refers to PINCS-1, a paired-sample study design postpartum comparing cervical screening performed at 6- or 12-weeks postnatal. The overall aim will be to assess the acceptability and feasibility of this study design in comparing conventional cervical screening and self-sampling at 6- versus 12-weeks postnatal."

There is redundancy in lines 3-10 and the primary study objective. Please

consider omitting or reformulate.

Aims and objectives/outcomes are subtly different – we have slightly reformulated.

Objective:

The primary objective will be, please change to past tense.

Thank you for the suggestions. We have changed to future tense and this is a protocol. Is that what you meant (see previous comment and your suggested edits)

Secondary objectives: 2) quality of clinician-collected LBC cervical samples? Please clarify, the

morphological quality.

- We have changed to clarify “To assess the quality of cervical samples from clinician-taken samples at 6-weeks postnatal through inadequacy rates.”
-

Methods and analysis:

Study design: omit to investigate etc as this is redundancy of the study objective. Just state the study

design and align it with the design written in the introduction, two-phase study with paired-sample

study design.

Thank you for this suggestion; we respectfully disagree.

Study setting: State the number of study sites.

Thank you for this suggestion. Amended as suggested.

Participants and recruitment:

State the timeline for the study inclusion.

Thank you for this suggestion. Amended as suggested.

Sample size:

Please insert references to the feasibility study that the experienced researchers have published.

This study was ongoing when we submitted the protocol in Aug 2024. Reference of pre-print now added.

Please consider omitting the considerations regarding the sample size of the PINCS2 study especially

as this will be reported in a separate study protocol paper.

Thank you for this suggestion. Amended as suggested.

Study visits:

- Please clarify that you applied co-testing with hrhPV testing and cytology for cervical scrapes and

using first-void urine samples. Please state if the 10 or 20-ml ColliPee device is used.

We have clarified this as suggested – co-testing is implied as performed at same visit prior to clinician-taken sample

Which hrHPV-test assay is used?

We have added : “. All urine samples will be tested at the cytology laboratory in Manchester using the Roche 8800 platform.”

Importantly, state the order of the cervical and FVU sampling, are

the cervical samples always taken first followed by the FVU samples? If so, I recommend the authors

to always start med the FVU collection as you risk several invalid HPV results for the FVU samples

due to the risk of blood following the cervical sampling.

Thank you we have added the clarification, as suggested.

- Has the patient questionnaire been properly validated?

Adapted from one in a previous study with PPIU feedback – added for clarification

Statistical analysis:

I would assume that in a study protocol paper, you would have decided on the final statistical

analysis plan.

As this is a feasibility study, this is not normal practice

Discussion:

The discussion is quite lengthy, please consider omitting some of the information about the

upcoming RCT trial, as this will be reported elsewhere.

Thank you

p .12, lines: 47-48. Also in Denmark, we have found that ethnic minority groups prefer urine over

vaginal sampling. You could consider stating that the finding in the YouScreen trial also is seen in

other countries including Denmark. Feel free to use the Danish reference, "Well, That Was Pretty

Clever!"-Ethnic Minority Women's Views on HPV Self-Sampling Devices for Cervical Cancer Screening: Attitudes to Brushes, First-Void Urine, and Menstrual Blood Devices"

We thank you for this information, which was similar to findings in the YouScreen study, which is already quoted. Thank you for this valuable information, which we can include in the subsequent results write up and will be helpful for the PhD literature review. We have been advised to limit references by the editorial team

- Another important outcome in this feasibility study and/or RCT trial should be the compliance to

follow-up among women who tested hrHPV positive in their self-sample. I think it is important to get

some perspectives from the women about this matter and not just if they will attend self-sampling 6

weeks postnatal. If high compliance to follow-up is not obtained when the self-sampling offer

provides more harm than preventive benefits. I miss this important perspective in the discussion.

Thank you for this comment – however, this is a feasibility study designed to see if patients will attend for paired samples (in a DTA study) and not one designed to look at uptake rates of subsequent compliance with screening – this is our ultimate intention but we need to show DTA first before we will be allowed to perform that study. We will collect data from subsequent colposcopy to inform the study, but this is not the primary or even secondary objective.

When writing that urine samples for hrHPV testing are under evaluation, please consider not just the

UK studies, but also the Belgian VALHUDES studies by Van Keer and colleagues.

Thank you we have added this reference.

3

What about the clinical management of the women, if the woman is HPV+ with HSIL cytology and

referred directly for colposcopy showing CIN3+, when will it then be ok to perform the LEETZ

procedure, etc? Do the authors have opinions about this matter?

The authors do have opinions, but this is not relevant to the protocol for a feasibility study of a subsequent DTA study, as explained in the discussion. This information is therefore outside of the scope of this paper/study.

Reviewer: 2
Dr. Eliane Rohner, University of Bern Institute of Social and Preventive Medicine
Comments to the Author:

Major comments

• The authors state that PINCS-2 will have its own study protocol. Nevertheless, they describe aspects like objectives or sample size considerations for PINCS-2 in the current study protocol. It has been quite difficult for me to disentangle which aspects will be part of PINCS-1 vs PINCS-2. I suggest that the authors refer all references to PINCS-2 into an outlook section.

Thank you for this suggestion. Amended as suggested.

• Do the authors plan to follow women over time to understand whether they will return for follow up assessments and treatment if needed?

Thank you for this suggestion. However, this is outside of the scope of the DTA feasibility study, which is not designed to look at uptake or subsequent management

• Could the authors elaborate more on why the decided to include women irrespective of their screening status? Are they not afraid that they might end up including a substantial proportion of women who do not require screening at this point (and might not be representative of the target population)?

Thank you for this suggestion. We have added our rationale for not doing this since the DTA study is not to test subsequent uptake of screening.

• Outcomes should be more clearly defined than just referring to them as binary variables.

Thank you for this suggestion. Additional information added.

• The authors should elaborate more on the planned statistical analysis, even if a detailed statistical analysis plan will follow later on.

Thank you for this suggestion. Additional information added.

• The authors mention diagnostic test accuracy (sensitivity and specificity), but what will be the definition of cervical disease? Cytological results? Histology?
Thank you for this suggestion. Additional information added.

Minor comments,

Abstract
• Please explain what the acronym PINCS stands for
Thank you for this suggestion. Amended as suggested.

Strengths and limitations
• "Not statistically powered to evaluate the diagnostic test accuracy" –diagnostic test accuracy is not listed as an objective of PINCS-1 anyway?
Indeed – although we note this study is larger than the one used to inform current UK guidance! Thank you for this suggestion. Amended for clarification.

Aim
• It would be helpful to remove the reference to PINCS-2 here, as it has the potential to confuse the reader
Thank you for this suggestion. Amended as suggested.

Objectives
• "The primary objective is to evaluate a paired-sample study design" – Is the objective to evaluate the study design or to assess feasibility?

Thank you for this suggestion. Amended as suggested.

• If I understand correctly, the justification for the repeat screening at 12 weeks postnatal is to assess women's willingness to undergo two cervical cancer screenings only 6 weeks apart to examine the feasibility of a future study which is supposed to examine the agreement between screening results at 6 and 12 months postnatal? It would be helpful if this was stated more clearly.
• 3rd secondary objective: do the authors plan to assess agreement between HPV status on cervical samples at 6 and 12 weeks, and the same for urine samples? Or are they interested in the agreement between the HPV status on urine and cervical samples? Are they referring to clinician- or self-collected cervical samples here?

All of the above

Methods
• Can the authors provide more information on the study setting and the study sites involved? For example, are these clinics or gynecological practices?

Thank you for this suggestion. Amended as suggested.

• How do the authors plan to handle abnormal screening results at 6 weeks? Figure 1 suggests that the 6-week sample will not be tested until the 12-week sample is obtained. Some of the information in figure 1 should be added to the text to help the reader understand the pathways better.

Thank you for this suggestion. Amended as suggested.

• Why did the authors decide to include a convenience sample rather than enrolling all eligible, consenting women who attend 6-week postnatal visits? How representative will this convenience sample be? Is this how they plan to enroll women for larger RCTs in the future? The implications of choosing a convenience sample should be discussed in the limitations section.

Future studies to determine the effect of change in practice will be performed in primary care. This is not required for DTA studies, where we are testing the accuracy of the sampling at each time point, not the uptake rate. Performing the small study in primary care would have required more resources and multiple sites.

We have changed limitations to:

"Limitations

- Screening will be performed in secondary care throughout this study, as this study is designed to test the feasibility of a future paired sample diagnostic test

accuracy study, not the effect on uptake in a primary care setting; this is a separate question, requiring different study design.

This will be discussed more fully in the final write up of the results.

We have also added:

"The study will be performed in secondary care, to limit number of sites required and control for variability of cervical sampling from multiple cervical screeners. This is because this study will examine the feasibility of a future large paired-sample study, comparing diagnostic test accuracy of cervical screening at 6- and 12-weeks postnatal. A different study design will be required in a further study to test the effect on uptake of cervical screening, if offered at the 6-week postnatal check-up. This further study will necessarily be conducted in primary care settings. However, we will need to confirm that this is safe and acceptable to the postnatal population before testing within the wider cervical screening programme."

• "... a cohort of 100 potential participates will be approached and the acceptance rate recorded" – how will participants recruited through leaflets and information events be considered in this?

This is a sub-study of approached participants only as we are unable to assess the acceptance rate through other recruitment methods

• Description of sample size calculation for PINCS-1 is rather vague.

Thank you for your comment. This is a feasibility study, designed to provide data for an a priori power calculation for a subsequent study, as explained.

• Sample size calculation for PINCS-2 should be removed from this protocol.

Thank you for this suggestion. Amended as suggested.

• "The study will consist of a screening and consent appointment" – I assume the authors are referring to eligibility screening, not cervical cancer screening here?

Thank you for this suggestion. Clarified as suggested.

• What is meant by "cervical sample/sweep test"? Is this a self-collected vaginal sample? What will they be used for in addition to the clinician-collected samples?

Thank you for this suggestion. Clarified as a clinician=taken cervical sample.

• Which HPV test will be used? Where will the laboratory tests be performed?

Information added

• "We will perform a patient questionnaire after sampling" – did the authors also consider administering a health care provider questionnaire to examine whether the providers experienced challenges incorporating cervical cancer screening into the 6 weeks postnatal appointment?

This will be part of a future primary care study but not applicable for this study. This question is the subject of a separate study.

• Figures 2 and 3: What do letters 1A, 2A etc refer to?

Sample letters added as supplementary information

VERSION 2 - REVIEW

Reviewer 2

Name Rohner, Eliane

Affiliation University of Bern Institute of Social and Preventive Medicine

Date 01-May-2025

COI

Thank you for addressing my comments. The only point that the authors did not fully address was the question regarding their definition of cervical disease (i.e., their reference/gold standard) for the calculation of sensitivity/specificity. In the response, they mentioned that they added additional information, but I could not find it? It would be useful if they adapted the following statement in the "statistical analysis" section to include a definition of their reference standard for the calculation of sensitivity an specificity: "We will use 2 x 2 tables to analyse sensitivity and specificity of: combination HPV testing and cytology of LBC samples at 6- and 12-weeks; HPV testing of LBC samples versus urine samples at both 6- and 12- weeks."
